# Urinary ATP May Be a Dynamic Biomarker of Detrusor Overactivity in Women with Overactive Bladder Syndrome

**DOI:** 10.1371/journal.pone.0064696

**Published:** 2013-05-31

**Authors:** Miguel Silva-Ramos, Isabel Silva, Olga Oliveira, Sónia Ferreira, Maria Júlia Reis, José Carlos Oliveira, Paulo Correia-de-Sá

**Affiliations:** 1 Laboratório de Farmacologia e Neurobiologia, UMIB, Instituto de Ciências Biomédicas Abel Salazar (ICBAS) - Universidade do Porto (UP), Porto, Portugal; 2 Serviço de Urologia - Centro Hospitalar do Porto (CHP), Porto, Portugal; 3 Serviço de Química Clínica - Centro Hospitalar do Porto (CHP), Porto, Portugal; IPO, Inst Port Oncology, Portugal

## Abstract

**Background:**

Nowadays, there is a considerable bulk of evidence showing that ATP has a prominent role in the regulation of human urinary bladder function and in the pathophysiology of detrusor overactivity. ATP mediates nonadrenergic-noncholinergic detrusor contractions in overactive bladders. *In vitro* studies have demonstrated that uroepithelial cells and cholinergic nerves from overactive human bladder samples (OAB) release more ATP than controls. Here, we compared the urinary ATP concentration in samples collected non-invasively from OAB women with detrusor overactivity and age-matched controls.

**Methods:**

Patients with neurologic diseases, history of malignancy, urinary tract infections or renal impairment (creatinine clearance <70 ml/min) were excluded. All patients completed a 3-day voiding diary, a 24 h urine collection and blood sampling to evaluate creatinine clearance. Urine samples collected during voluntary voids were immediately freeze-preserved for ATP determination by the luciferin-luciferase bioluminescence assay; for comparison purposes, samples were also tested for urinary nerve growth factor (NGF) by ELISA.

**Results:**

The urinary content of ATP, but not of NGF, normalized to patients’ urine creatinine levels (ATP/Cr) or urinary volume (ATP.Vol) were significantly (*P*<0.05) higher in OAB women with detrusor overactivity (*n* = 34) than in healthy controls (*n* = 30). Significant differences between the two groups were still observed by boosting urinary ATP/Cr content after water intake, but these were not detected for NGF/Cr. In OAB patients, urinary ATP/Cr levels correlated inversely with mean voided volumes determined in a 3-day voiding diary.

**Conclusion:**

A high area under the receiver operator characteristics (ROC) curve (0.741; 95% CI 0.62–0.86; *P*<0.001) is consistent with urinary ATP/Cr being a highly sensitive dynamic biomarker for assessing detrusor overactivity in women with OAB syndrome.

## Introduction

Overactive bladder (OAB) is a complex clinical syndrome based on self-reported symptoms of urinary urgency associated with incontinence in up to one-third of cases, usually accompanied by daytime frequency and nocturia, in the absence of proven infection or other obvious pathology [1]. It has been reported an overall prevalence of 12,8% in women [2], and a significant impact on patients quality of life [3]. The precise pathogenesis underlying OAB remains to be clarified and might be multifactorial. OAB symptoms are often associated with detrusor overactivity, which is diagnosed by invasive urodynamic testing as involuntary contractions of the detrusor muscle during bladder filling. Regrettably, the relationship between clinical symptoms and urodynamic findings is not reliable [4], [5]. In fact, procedure constrains such as saline infusion rate and temperature, patient position, and anxiety, may yield distinct urodynamic results [6], which makes urodynamic confirmation of detrusor overactivity with only limited clinical value regarding the severity or prognosis of idiopathic OAB [7], [8]. This renders urodynamic investigation with suboptimal characteristics to evaluate OAB patients and urge the search for new accurate, reliable and non-invasive tests to predict detrusor overactivity and to assess patients’ therapeutic outcome (reviewed in [9]).

Recently, low grade inflammatory mediators, such as cytokines, prostaglandin E_2_ (PGE_2_) and nerve growth factor (NGF), gained significant attention as urinary biomarkers of detrusor overactivity. Although they correlate with OAB symptom severity, they have not been shown to have independent prognostic benefit (reviewed in [10]) and they still cannot replace the standard filling cystometry in standard clinical practice [9]. For instance, pilot studies indicate that there is a wide variation of urinary NGF levels and not all patients with OAB have elevated urinary NGF [11]–[13].

Purinergic transmission has increasingly been accepted as having an important role in urinary tract dysfunction. It encompasses both efferent and afferent paths of the voiding reflex, and has particular relevance in the pathogenesis of detrusor overactivity [14], [15]. Several studies have reported that urothelium releases ATP in response to mechanical and chemical stimuli [16], [17]. These stimuli evoke a discharge in suburothelial low-threshold sensory nerves through P2X3 receptors activation, since sensory nerve excitation is substantially reduced by P2X3 antagonists [18] and in P2X3 knock-out mice [19], also the density of these receptors is increased in patients with detrusor overactivity [20]. Conversely, intravesical instillation of ATP increases bladder activity in a concentration dependent manner [21]; intravesical ATP decreases bladder capacity and micturition volume, with limited effect on detrusor pressure. Recently, evidence has been emerging in support of a role for suburothelial myofibroblasts in detrusor contraction, which also express P2X and P2Y purinoceptors [22]. In detrusor smooth muscle fibres, P2X1 receptor subtype expression is also markedly increased in unstable bladders (reviewed in [15]). This implies that ATP is an important mediator of bladder sensory/effector pathways.

Increased ATP release from the urothelium has also been demonstrated in animal models of OAB [23]–[25]. *In vitro* studies conducted in our lab, as well as by others, showed that uroepithelial cells and cholinergic nerves from overactive or obstructed human bladder samples release more ATP than controls [26], [27]. Interestingly, strong evidences suggest that the primary source of ATP in the human bladder is the urothelium [28]. Thus, this study was prospectively designed to measure the urinary concentration of ATP in OAB women tested positively for detrusor overactivity and in age-matched controls to assess the putative role of ATP as a non-invasive biomarker of detrusor overactivity in OAB patients. For comparison purposes, urine samples were also tested for NGF levels.

## Methods

### Patients and Procedures

**Figure 1:**
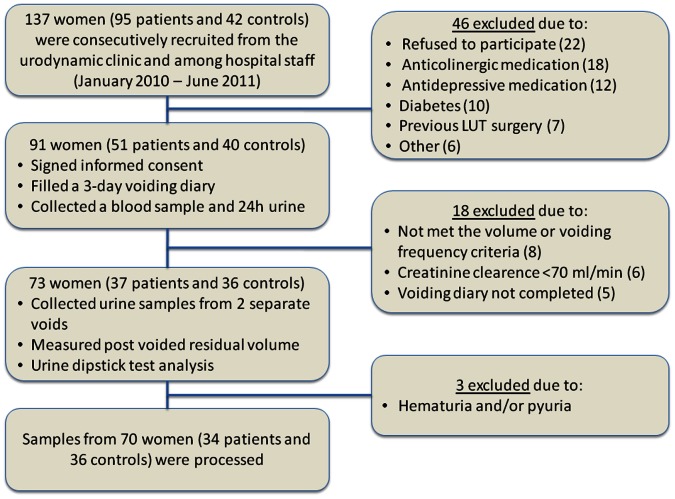
Flow diagram of patients’ selection.

This study and all its procedures were approved by the Ethics Committees of Centro Hospitalar do Porto (CHP) and of Instituto de Ciências Biomédicas de Abel Salazar (Medical School) of the University of Porto (ICBAS-UP). All patients signed an informed consent prior to examinations and for using the biological material. The investigation conforms to the principles outlined in the Declaration of Helsinki. A total of 70 women were enrolled in this study, including 34 patients with overactive bladder symptoms and detrusor overactivity (aged 28–84 years, mean 57±13 years) and 36 controls (aged 34–67 years, mean 52±9 years) that included asymptomatic volunteers recruited among the hospital staff and patients with mild stress incontinence without overactive bladder symptoms and normal urodynamics (see Figure 1). This was a prospective study. Patients were consecutively selected (January 2010–June 2011) from outpatients consulting the Department of Urology of CHP complaining from urgency (at least one episode daily), frequency (>8 voids/day) and reduced bladder capacity (mean voided volume <300 ml). Detrusor overactivity was confirmed in all women with OAB by urodynamic testing performed within 12 months before collection of biological samples and the results were analysed in accordance to the criteria of the International Continence Society (ICS) defining detrusor overactivity as involuntary and spontaneous contractions of the detrusor occurring during the storage phase. Patients with history of malignancy, pelvic radiotherapy, neurologic disease, any systemic or inflammatory condition, active urinary tract infections, renal impairment (creatinine clearance <70 ml/min) or taking any medication that could affect bladder function within the 14 days time period before sample collection were excluded; pregnant women were also excluded from the study. Patients with incomplete emptying (post-void residual volume >100 ml) and with voiding dysfunction (Pdet at maximal flow >60 mm H_2_O) were also not included in the study. All patients completed a 3-day voiding diary, a 24 h urine collection and blood sample collection to estimate creatinine clearance. Subjects were asked to void at normal desire into a sterile cup. Thus, urine sample collection in this work was non-invasive, which contrasts with previous studies that used urodynamic fluid voided after cystometric saline bladder filling [29], [30]. Two consecutive voids were used; the first void was collected upon patients’ arrival to consultation and the second void was collected after allowing patients to drink 500 mL of water *ad libitum*. Voided volume was recorded and post-void residual volume was estimated by ultrasonography. Mid-stream urine samples were divided into three tubes; one for sediment examination, another for creatinine measurement and a third was immediately freeze-dried in liquid nitrogen and preserved at −80°C until ATP determination. ATP values were normalized according to urinary creatinine (Cr) values to reduce sample variation and to discard kidney influence on ATP levels. ATP/Cr ratios were compared between patients and controls.

### Measurement of Urinary Creatinine

The quantitative determination of urinary creatinine was performed *in vitro* on a Cobas Integra 800 analyser using the kinetic colorimetric Creatinine Jaffé Gen.2 assay according to the manufacturers’ instructions (Roche Diagnostics GmbH, Mannheim, Germany).

### Measurement of Urinary ATP

Undiluted urine samples were defrosted till 25°C and afterwards centrifuged at 3000 g at room temperature for 20 seconds to remove cellular debris. The supernatant was separated. A mixture of luciferine-luciferase was added according to the manufacturer instructions using the ATP Bioluminescence Assay Kit HS II (Roche Applied Science Indianapolis, Indiana, USA). ATP detection was evaluated using a multi-mode microplate reader (Synergy HT, BioTek Instruments Inc., Vermont, USA) controlled via BioTek’s Gen5™ Data Analysis Software. Sample bioluminescence was compared to that of standard amounts of ATP used in the same concentration range; standard ATP samples were prepared daily. All samples were run in duplicate. The minimum ATP detection limit in these experimental conditions was 10^−12^ M (10^−16^ moles in 100 µl samples) and luminescence correlated linearly to ATP concentration till 10^−6^ M. Each urine sample remaining was used to quantify the lactate dehydrogenase (LDH, EC 1.1.1.27) activity [31]. LDH is an intracellular enzyme which is commonly used as an indicator of cell integrity providing that its values are kept at a low level.

### Measurement of Urinary NGF

Measurement of urinary NGF level was also done in a subset of urine samples used for ATP determination (technique reviewed by [11]). After defrosting and homogenization, samples were used to measure NGF levels by the ELISA method, according to the manufacturer instructions. We determined urinary NGF concentration using the E_max_ ImmunoAssay System (Promega, Madison, EUA), a specific ELISA kit that has a minimum sensitivity of 7.8 pg/ml. All samples were run in duplicate. NGF values were also normalized according to urinary creatinine (Cr) values to reduce sample variation; obtained NGF/Cr ratios were compared between patients and controls.

Routine measurements of blood and urine samples were performed in a blind manner by independent observers from Serviço de Química Clínica - Centro Hospitalar do Porto (CHP, Porto). Urinary ATP and NGF contents were tested in blind samples at Laboratório de Farmacologia e Neurobiologia (UMIB/ICBAS-UP). NGF measurements were re-tested and confirmed with the methodology in use at the Department of Urology, Centro Hospitalar de S. João and Faculty of Medicine, University of Porto.

### Statistical Analysis

Statistical analyses were performed using GraphPad Prism 5.04 software (La Jolla, USA). Results are reported as mean values ± standard deviation (SD) of samples collected during the first void, unless stated otherwise. Kolmogorov-Smirnov test was used to check for normality of data distribution. Unpaired Student’s *t*-test with Welch’s correction and Mann-Whitney U-test were used for statistical analysis between groups when parametric or nonparametric data was considered, respectively. For multiple comparisons, one-way ANOVA nonparametric Kruskal-Wallis test with Dunn’s post test modification was used. Correlation between variables were analysed using the Spearman test. *P*<0.05 (two-tailed) values were considered statistically significant.

## Results

In this study, efforts have been made to match age among OAB women with detrusor overactivity and the control group (*P*>0.05, see Table 1), since it has been described that urinary ATP may increase in older subjects [32]; see in Table 2 the positive correlation between age and ATP/Cr levels found in our series (Spearman test, r = 0.380, *P*<0.05). The two groups were also essentially similar (*P*>0.05) regarding creatinine clearance, urine pH and urinary LDH activity (Table 1). Early reports indicate that females with detrusor overactivity may have significantly lower urinary pH than controls [33]. This tendency did not reach statistical significance (*P*>0.05) in this series excluding any possible influence the pH might have on urinary ATP and NGF content in control and OAB patients. As expected, maximum and mean voided volumes determined from the 3-day voiding diary were significantly (*P*<0.05) lower in OAB patients than in controls (Table 1).

**Table 1:** Characteristics of OAB patients and controls. *P*<0.05 (unpaired Student’s *t*-test with Welch’s correction) represent significant differences from controls.

	Controls (n = 36)mean±SD	OAB (n = 34)mean±SD	*P*
Age (yrs)	51.8±8.7	57.3±12.8	0.055
Creatinine clearance (ml/min)	98.9±9.6	116.7±7.1	0.171
Urinary pH	6.7±0.4	5.9±0.3	0.135
Urinary LDH (U/ml)	12.1±3.7	6.8±1.2	0.174
*3-Day Voiding Diary:*			
Minimum Void Volume (ml)	86.6±21.3	73.7±23.5	0.263
Maximum Void Volume (ml)	425.0±121.5	326.1±85.3	**0.009**
Mean Void Volume (ml)	198.6±31.1	159.9±40.2	**0.033**
*1^st^ Void (upon arrival):*			
Vol. 1^st^ Void (ml)	203.3±56.5	210.8±61.7	0.810
Urinary ATP 1^st^ Void (pM)	2647±403	7004±1228	**0.001**
Urinary ATP x Vol. 1^st^ Void (pmol)	707.8±139.6	1556.1±290.9	**0.012**
Urinary ATP/Cr 1^st^ Void (pmol/mg)	7.2±1.7	27.5±8.3	**0.022**
Urinary NGF 1^st^ Void (pg/ml)	530.5±136.2	467.5±193.0	0.397
Urinary NGF x Vol. 1^st^ Void (ng)	117.9±42.3	67.5±15.7	0.126
Urinary NGF/Cr 1^st^ Void (pg/mg)	64.0±13.6	109.5±29.0	0.162
*2^nd^ Void (after water intake):*			
Volume of 2^nd^ Void (ml)	310.0±77.5	260.9±66.9	0.181
Urinary ATP 2^nd^ Void (pM)	5700±978	10577±1533	**0.006**
Urinary ATP x Vol. 2^nd^ Void (pmol)	2003.1±366.4	3008.4±517.5	**0.022**
Urinary ATP/Cr 2^nd^ Void (pmol/mg)	42.5±7.2	89.1±19.3	**0.029**
Urinary NGF 2^nd^ Void (pg/ml)	526.0±227.1	296.2±128.4	0.185
Urinary NGF x Vol. 2^nd^ Void (ng)	82.7±24.0	39.2±10.4	**(0.045)**
Urinary NGF/Cr 2^nd^ Void (pg/mg)	86.0±17.4	121.7±33.8	0.352

**Table 2:** Correlation between variables analysed using the Spearman test. *P*<0.05 values were considered statistically significant.

	Controlsr (Spearman approx.)	*P*value	OAB Patientsr (Spearman approx.)	*P*value
ATP/Cr vs NGF/Cr	0.378	**0.023**	0.221	0.118
ATP/Cr (1^st^ *vs* 2^nd^ voids)	0.774	**<0.0001**	0.381	**0.013**
NGF/Cr (1^st^ *vs* 2^nd^ voids)	−0.109	0.291	0.307	**0.046**
Age *vs* ATP/Cr	0.380	**0.019**	−0.198	0.131
Age vs NGF/Cr	0.217	0.134	−0.115	0.165
LDH vs ATP/Cr	−0.054	0.399	0.249	0.085
LDH vs NGF/Cr	−0.185	0.199	−0.450	**0.006**

**Figure 2:**
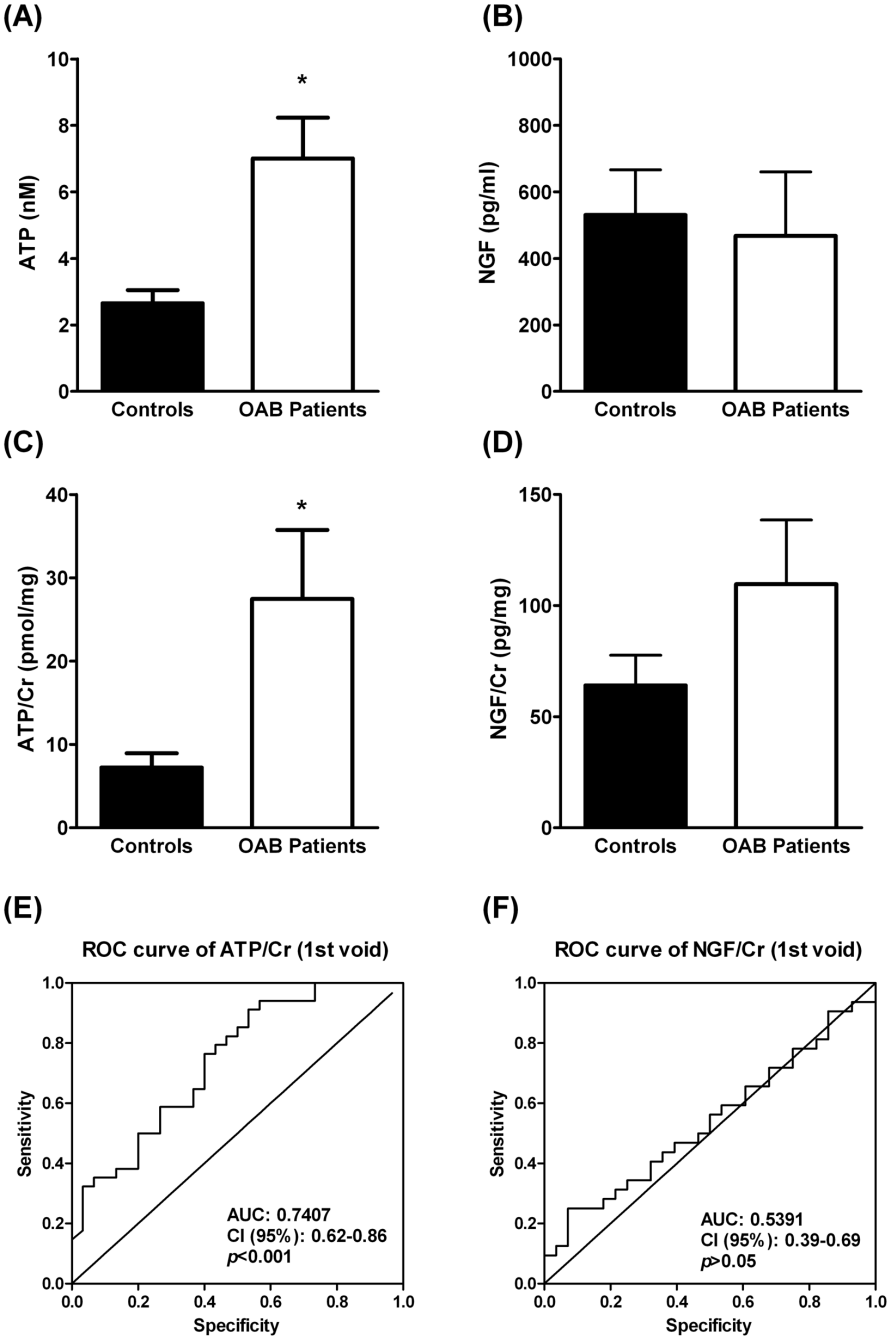
Urinary ATP and NGF levels determined before (A: ATP, nM and B: NGF, pg/ml) and after normalization to urine creatinine content (C: ATP/Cr, pmol/mg and D: NGF, pg/mg) in women with OAB (n = 34) and age-matched controls (n = 36). Columns are mean values ± standard deviation (SD). *P<0.05 (unpaired Student’s *t*-test with Welch’s correction) represent significant differences from controls. In E and F, represented are the receiver-operator characteristics (ROC) curves of urinary ATP/Cr and urinary NGF/Cr in OAB patients, respectively. Please note that, for this cohort, ATP/Cr has a better area under de curve (AUC = 0.7407; 95% confidence interval (CI) = 0.62–0.86; *P*<0.001) than NGF/Cr (AUC = 0.5391; 95% CI = 0.39–0.69; *P*<0.001).

Urinary ATP levels were significantly (*P*<0.05) higher in OAB women with detrusor overactivity as compared to the control group (Table 1 and Figure 2A). This was also verified when the ATP content was normalized either to urine creatinine levels (ATP/Cr, pmol/mg) (Figure 2C) or to the urinary volume (ATP.Vol, pmol) (Table 1); the latter reflects better the raw amount of ATP accumulated in the bladder during urine storage. Since no significant changes (*P*>0.05) were observed in the activity of the intracellular enzyme, LDH, in urine samples from OAB patients and age-matched controls (Table 1) and no correlation was found between ATP/Cr levels and LDH activity (Table 2), urinary cells damage might not account for changes detected in ATP levels in OAB patients and controls.

The Receiver Operating Characteristics (ROC) graphs are commonly used in medical decision making. ROC graphs have long been used in signal detection theory to depict the tradeoff between hit rates and false alarm rates of classifiers [34]. A high area under the ROC curve (0.741; 95% CI 0.62 to 0.86; *P*<0.001) is consistent with urinary ATP/Cr being a highly sensitive biomarker for discriminating OAB women with detrusor overactivity (Figure 2E).

**Figure 3:**
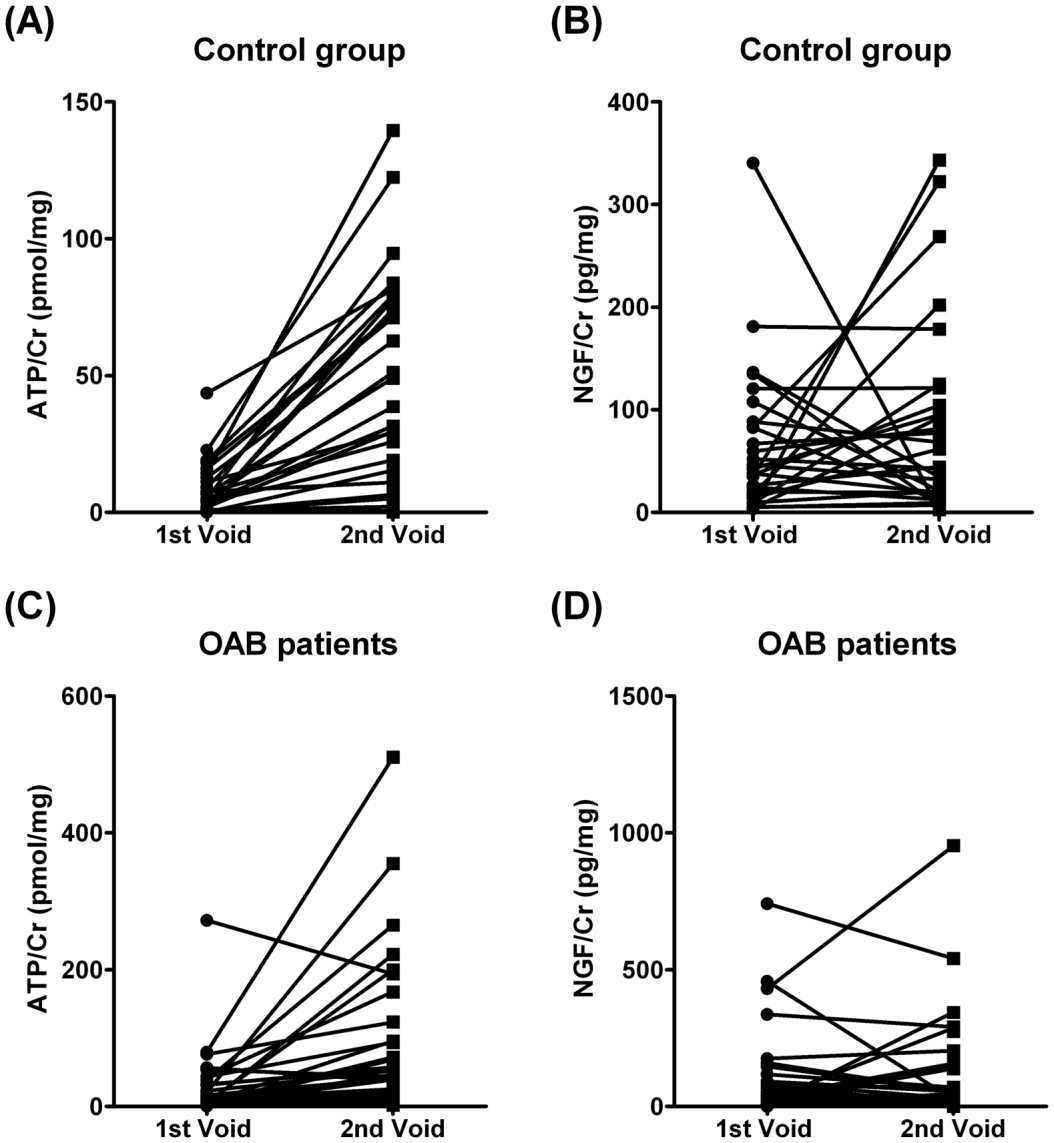
Analysis of ATP/Cr (pmol/mg) and NGF/Cr (pg/mg) content in paired urine samples collected from control women (A and B) and OAB patients (C and D) at arrival to the consultation (1^st^ void) and after water intake (2^nd^ void). See Table 2, for correlation between variables (Spearman test).

Although NGF levels normalized to creatinine content in the urine (NGF/Cr, pg/mg) tended to increase in OAB patients as compared to controls, this trend did not reach statistical significance (*P*>0.05) (Figure 2D, see also [35]; *cf.* [36]). We found no significant correlation between ATP/Cr and NGF/Cr levels measured in parallel from samples collected from OAB patients (Spearman test, r = 0.221, *P*>0.05), despite the two compounds showed some degree of correlation in the control group (Table 2). We did not found statistically significant differences in the concentration of NGF (pg/ml) measured in urine samples from control and OAB patients (Figure 2B) and the urinary NGF accumulation during bladder storage normalized to the urinary volume (NGF.Vol, ng) was even lower in OAB patients than in controls (Table 1). We detected a wide variation (and lack of correlation) of urinary NGF/Cr levels in paired samples collected from control individuals between the 1^st^ and the 2^nd^ void obtained after water intake, which was no better in women with OAB (Figure 3B and 3D, see also Table 2). These results suggest that NGF determination is highly dependent on the urinary volume (Table 1), a situation that has not been reported in previous studies. The statistical analysis presented in Table 1, shows that the mean urinary NGF content normalized to voided volume decreased significantly in OAB patients as compared to healthy controls as a consequence of forcing the desire to void by water intake (2^nd^ void). This finding is in clear contrast to that occurring with urinary ATP; the urinary content of the nucleotide boosted in the 2^nd^ void upon increasing the urinary volume by drinking water *at libitum* (for correlation analysis, see Table 2). It is worth noting that water intake (after 1^st^ void) increases the sensitivity of urinary ATP determinations in OAB patients *versus* controls (Table 1, ROC curve area = 0.769; 95% CI = 0.64–0.90; *P*<0.001).

**Figure 4:**
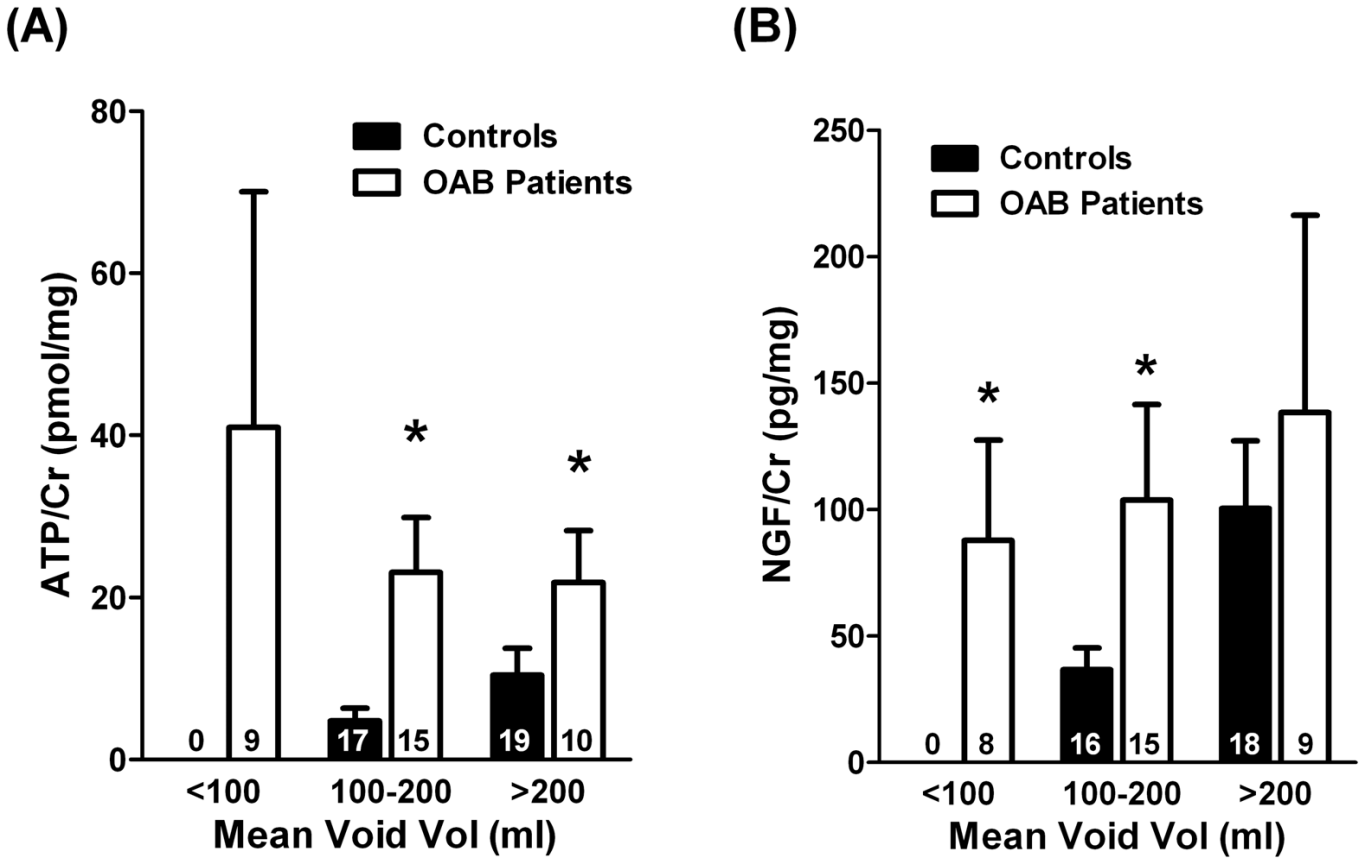
Relationship between urinary (A) ATP/Cr (pmol/mg) and (B) NGF/Cr (pg/mg) content and the mean voided volume determined in a 3-day voiding diary for women with OAB and age-matched controls. Columns are mean values ± standard deviation (SD) from *n* number of individuals (shown in parenthesis). *P<0.05 (unpaired Student’s *t*-test with Welch’s correction) represent significant differences from controls.

Figure 4A, shows that urinary ATP/Cr levels correlate inversely with the mean voided volume determined in a 3-day voiding diary in OAB patients. In other words, urinary ATP/Cr was maximal in OAB patients who reported a mean voided volume less than 100 ml (observed in 9 out of a total of 34 patients). These findings contrast with data from the control group where ATP levels normalized to urine creatinine content (ATP/Cr, pmol/mg) positively correlates with the mean voided volume (Spearman test, r = 0.522, *P*<0.01) (see Figure 4A) and fully agree with the concept that physiological distension of the urothelium releases ATP into the lumen of the bladder [16], [17]. No other parameter analyzed in this study changed significantly (*P*>0.05) between the three subgroups identified using the mean voided volume as criteria, namely age and urine measurements of creatinine, pH and LDH activity. Thus, finding a correlation between ATP/Cr levels and the mean voiding volume may be taken as a marker for the severity of detrusor overactivity and should be considered of clinical relevance in the follow-up of OAB syndrome. Using 200 ml as a cutoff value, we found that urinary NGF/Cr levels in OAB patients were significantly (*P*<0.05) higher than those found in the control group, but this difference disappeared in less severe forms of bladder overactivity (Figure 4B).

**Figure 5:**
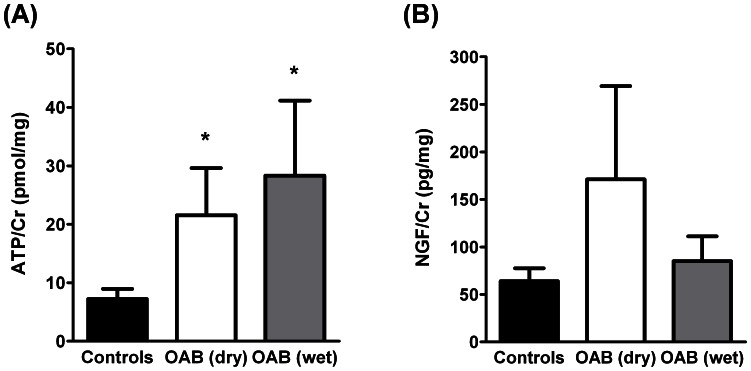
Urinary (A) ATP/Cr (pmol/mg) and (B) NGF/Cr (pg/mg) content in women with detrusor overactivity complaining only of urgency (OAB dry, n = 7) and of urgency associated with urge urinary incontinence (OAB wet, n = 21) as compared to age-matched controls (n = 36). Columns are mean values ± standard deviation (SD). *,**P<0.05 (one-way ANOVA with Dunn’s multiple comparison test) represent significant differences from controls and between OAB dry and OAB wet subgroups, respectively.

In this series, 25% of the women with detrusor overactivity complained of urgency without incontinence (OAB dry), whereas the remaining group (75%) had urgency urinary incontinence (OAB wet). Figure 5 shows that urinary ATP/Cr, but not NGF/Cr, levels were significantly (*P*<0.05) higher than controls in women with OAB, despite they have been characterized as OAB dry or OAB wet. No significant differences (*P*>0.05) were, however, detected in urinary ATP/Cr and NGF/Cr levels among OAB patients of the two subcategories.

## Discussion

To our knowledge, this is the first study so far designed to evaluate synchronous changes of both ATP and NGF levels in urine samples collected, non-invasively, during voiding at normal desire by OAB women with detrusor overactivity and age-matched controls. Data shows that ATP levels were significantly higher in OAB women with detrusor overactivity than in the control group and this was observed when ATP levels were normalized either to urine creatinine content (ATP/Cr, pmol/mg) or to the urinary volume (ATP.Vol, pmol). A high area under the ROC curve (0.741; 95% CI 0.62–0.86; *P*<0.001) indicates that urinary ATP/Cr may be a highly sensitive biomarker for discriminating OAB women with detrusor overactivity. In this series, we failed to obtain a similar pattern regarding urinary NGF measurements. Although we found a tendency for NGF normalized to urine creatinine content (NGF/Cr) to increase in OAB patients as compared to controls, this tendency did not reached statistical significance (*P*>0.05). Moreover, no significant correlation between urinary ATP/Cr and NGF/Cr levels was found in the samples collected from OAB women with detrusor overactivity.

Interestingly, ATP levels measured in paired samples collected after water intake (2^nd^ void) consistently exceeded those verified in the samples collected upon arrival to the consultation (1^st^ void), both in controls and OAB patients. That is, for a given patient or control, ATP/Cr content in the 2^nd^ void boosted and significantly correlated with the amount detected in the previous (1^st^) void. This situation was clearly different if one considers NGF/Cr measurements. In keeping with the assumption that urinary NGF content is sensitive to urinary volume variations, we found a wide dispersion between measurements of NGF in paired samples obtained from 1^st^ and 2^nd^ voids, both in controls and in patients with OAB. Our findings fully agree with *in vitro* experiments demonstrating that mechanical distension of bladder urothelium from patients with OAB release significantly more ATP than controls [26]. Thus, water intake may increase the sensitivity of urinary ATP measurement as a biomarker of detrusor overactivity in OAB patients (ROC curve area = 0.769; 95% CI = 0.64–0.90; *P*<0.001). Given that the mean voided volume during the provocative 2^nd^ void significantly increases with respect to the 1^st^ void one would expect that the concentration of the nucleotide should dilute accordingly, yet this was not the case probably because raw ATP production by bladder distension exceeds the water dilution factor, particularly in OAB patients. This was verified even though urinary ATP measured in this study might have been underestimated due to the hydrolysis of the nucleotide by ecto-NTPDases bound to urothelial cells during urine bladder storage. Thus, the more frequent the voids (e.g. OAB patients with detrusor overactivity) the higher urinary ATP levels should be measured. Despite this, we measured higher than control levels of ATP in urine samples collected from OAB patients upon arrival to the consultation (1^st^ void) where no significant differences were detected in the voided volume and inferred time to void (see Table 1). ATP may, thus, be regarded as a novel and important dynamic urinary biomarker of detrusor overactivity which levels reflect adequately bladder distension under pathological conditions.

Several mechanisms may account for increased ATP levels in the urine of OAB women with detrusor overactivity. Hypertrophy and hyperplasia of the urothelium and underlying smooth muscle layers are frequently observed in overactive bladders. Thus, OAB patients might have increased urinary ATP levels produced by thickening of the epithelium taking into account that uro-epithelium is in close contact with the urine and is a major source of the nucleotide in the bladder [26], [27]. Whether urothelial cells *per se* release more ATP in OAB patients than in healthy controls remains to be determined. In addition, it is known that parasympathetic nerves from overactive or obstructed human bladder strips release more ATP than controls [15], [26], [27]. Enhanced urinary ATP content may also result from impairment of ecto-NTPDase activity observed in cells from OAB patients, as this finding may determine slower nucleotide inactivation kinetics in the bladder wall and its urinary accumulation [37], [38].

Data from this study demonstrate that urinary ATP/Cr levels correlated inversely with the mean voided volume obtained in a 3-day voiding diary applied to OAB patients. Although the urinary content of the nucleotide corrected to the creatinine level (ATP/Cr) was consistently higher in samples from OAB patients than in age-matched controls, differences tend to decrease when the clinical condition of the patients was less severe (*e.g.* mean voided volume higher than 200 ml). This tendency fully agrees with the lack of differences in the concentration of ATP released during cystometric bladder filling in women with mild bladder pain syndrome and controls [30]. Previous reports also demonstrated an inverse correlation between first desire to void and ATP concentration in the voided urodynamic fluid collected from OAB patients during cystometry investigation [29]. Both findings are consistent with the hypothesis that ATP has a prominent role modulating the early filling sensation in patients with OAB, which yields to low bladder volumes at first desire to void and symptoms of urgency, frequency and reduced bladder capacity presented by patients of this series. Thus, higher concentrations of ATP would produce early voiding reflexes in OAB patients as suggested in this study. Recently, Sugaya and collaborators [32] investigated the urinary ATP/Cr ratio in a population of 30 women with OAB (aged 69±8 years old) and 6 younger female controls (aged 37±6 years old). The ATP/Cr ratio in the Sugaya’s study was about one log unit lower than that obtained in our work, probably because cryopreservation of urine samples was not undertaken before readings. These authors did not found statistically significant differences in the urinary ATP content between the two groups, yet patients with higher ATP/Cr ratio had more severe symptoms and worse quality of life scores than the group exhibiting lower urinary ATP content, which fully agrees with our findings. Moreover, they reported that ATP/Cr levels significantly decreased after treatment with anti-muscarinic drugs and the improvement of lower urinary tract symptoms was greater for patients with a high baseline urinary ATP/Cr level [32]. These findings suggest that urinary ATP may be a marker of disease severity and might also be used to predict response to clinical intervention. Preliminary results from our group indicate that symptoms relief after intravesical botulinum toxin type A application in six female patients with detrusor overactivity of this series was accompanied by marked improvement of lower urinary tract symptoms along with a complete regression of urinary ATP/Cr to control levels (unpublished observations).

By far the most studied biomarker of OAB has been NGF, as this neurotrophin is highly expressed in the urothelium and suburothelial region of patients with bladder dysfunction (reviewed in [11], [13], [36]). There is some evidence from these pilot clinical studies using small patient populations that urinary NGF excretion is also elevated in patients with OAB. This tendency was also observed in this series, yet statistical significance was only obtained for women with the most severe forms of bladder overactivity reporting mean urinary volumes lower than 200 ml in the 3-day voiding diary. However, the peptide may be a downstream element of several bladder pathologies that may occur without urinary urgency (*e.g.* bladder inflammation, outlet obstruction, urolithiasis, neoplasia) (reviewed in [11], [13]). This may cause urinary NGF concentration to vary considerably amongst patients with similar complaints, yet it may still find some utility to assess OAB management as urinary NGF decreased after non-pharmacological and pharmacological treatments (reviewed in [11], [36]). Preliminary results published in the literature showed a superior accuracy of brain derived neurotrophic factor (BDNF) over NGF in diagnosing detrusor overactivity [36]. In contrast to NGF, a direct release of BDNF from urothelial or smooth muscle cells was never demonstrated and one cannot ignore that other organs besides the bladder may also release neurotrophic factors [39], [40]. Before adopting neurotrophins as urinary biomarkers of detrusor overactivity, one must also consider several limitations, in addition to the general lack of information about the levels of urinary neurotrophins in normal individuals. Methodological limitations include (a) the variation of urinary NGF and BDNF measurements by enzyme-linked immunoabsorbent assay (ELISA), as this method is not routinely available and requires technical expertise; (b) the binding of neurotrophins to proteins or cells potentially present in the urine of patients; and (c) the specificity of various antibodies against NGF and BDNF precursors, which may add variability to measured urinary neurotrophins. In addition, the reported dependence of urinary NGF content on variations of the voided volume (this study) and bladder distension [11], as well as the lack of studies indicating that there is a clear-cut clinical correlation between neurotrophin levels in urine samples and the severity of detrusor overactivity, may contribute to question their sensitivity to identify individuals with specific bladder pathologies or patients with detrusor overactivity.

In this regard, ATP has a very simple, inexpensive and highly reliable measurement method, the luciferin-luciferase bioluminescence assay. Technical limitation relies on the need for snap-freeze urine samples immediately after collection to avoid ATP degradation during storage. Moreover, urinary ATP is influenced by bacterial infection, kidney dysfunction and malignancy, so care must be taken to exclude such factors. In contrast to neurotrophins, differences between OAB patients and controls are independent on the urinary volume and, in fact, sensitivity of urinary ATP measurements in OAB patients may be enhanced by urine production and consequent bladder filling by water intake. This study proved that there is a positive correlation between urinary ATP levels and clinical symptoms evaluated by simply adding a 3-day voiding diary to analytical tests. Surprisingly or not, we found no statistical significant (*P*>0.05) differences between urinary ATP/Cr levels in OAB patients complaining of urgency without incontinence (OAB dry) and those reporting incontinence urinary urgency (OAB wet). This supports the notion that incontinence is inconsistently correlated and may have a pathophysiological mechanism distinct from detrusor overactivity, which might be independent from ATP bladder production. Because several mechanisms may be involved in the pathophysiology of the OAB syndrome, one cannot exclude the benefit of using a panel of urinary biomarkers (including neurotrophins) to target detrusor overactivity and intervention follow-up. Unfortunately, we found no correlation between ATP/Cr and NGF/Cr levels in urine samples collected from woman with OAB in this series.

In conclusion, our results support an important putative role of ATP in the pathogenesis of detrusor overactivity. Data showed that OAB women with detrusor overactivity have high urinary levels of ATP compared to controls and that the nucleotide levels increase with water intake. The increased concentration of ATP in the urine of OAB patients is probably due to enhanced ATP release from proliferating urothelium, since uroepithelial cells are considered the primary source of ATP in the human bladder [28]. This hypothesis agrees with previous studies using experimental models of bladder disorders exhibiting enhanced release of ATP from the urothelium [41], [42]. Moreover, *in vitro* studies demonstrated that uroepithelial cells from overactive human bladder samples release more ATP than controls and that the release of the nucleotide was resistant to blockade of neuronal activity with tetrodotoxin [26], [27]. Decreased ATP metabolism could also explain high levels of ATP in the urine of OAB patients as compared to controls. Reports from our and others groups, have shown that ATP catabolism is hindered in the bladder of patients with outlet obstruction and detrusor overactivity [27], [37], [38], which could also be the case of women with OAB. Thus, our findings are consistent with urinary ATP being a putative dynamic biomarker of detrusor overactivity in women with OAB. Non-invasive urinary ATP measurements may represent an improvement from other diagnostic and follow-up approaches requiring urodynamic investigations. Future studies are needed to further examine whether urinary ATP can also be used as a prognostic factor and to assess the therapeutic outcome in OAB women with detrusor overactivity.
